# Quantification of Cas9 binding and cleavage across diverse guide sequences maps landscapes of target engagement

**DOI:** 10.1126/sciadv.abe5496

**Published:** 2021-02-19

**Authors:** Evan A. Boyle, Winston R. Becker, Hua B. Bai, Janice S. Chen, Jennifer A. Doudna, William J. Greenleaf

**Affiliations:** 1 Department of Genetics, Stanford University School of Medicine, Stanford, CA 94305, USA.; 2 Program in Biophysics, Stanford University, Stanford, CA 94305, USA.; 3 Department of Molecular and Cell Biology, California Institute for Quantitative Biosciences (QB3), University of California, Howard Hughes Medical Institute, Department of Chemistry, and the Innovative Genomics Institute, University of California, Berkeley, Berkeley, CA 94720, USA.; 4 MBIB Division, Lawrence Berkeley National Laboratory, Berkeley, CA 94710, USA.; 5 Gladstone Institutes, University of California, San Francisco, San Francisco, CA 94158, USA.; 6 Department of Applied Physics, Stanford University, Stanford, CA 94305, USA.

## Abstract

The RNA-guided nuclease Cas9 has unlocked powerful methods for perturbing both the genome through targeted DNA cleavage and the regulome through targeted DNA binding, but limited biochemical data have hampered efforts to quantitatively model sequence perturbation of target binding and cleavage across diverse guide sequences. We present scalable, sequencing-based platforms for high-throughput filter binding and cleavage and then perform 62,444 quantitative binding and cleavage assays on 35,047 on- and off-target DNA sequences across 90 Cas9 ribonucleoproteins (RNPs) loaded with distinct guide RNAs. We observe that binding and cleavage efficacy, as well as specificity, vary substantially across RNPs; canonically studied guides often have atypically high specificity; sequence context surrounding the target modulates Cas9 on-rate; and Cas9 RNPs may sequester targets in nonproductive states that contribute to “proofreading” capability. Lastly, we distill our findings into an interpretable biophysical model that predicts changes in binding and cleavage for diverse target sequence perturbations.

## Teaser

A massive new resource of diverse Cas9 binding and cleavage events establishes new rules for effective CRISPR targeting.

## INTRODUCTION

*Streptococcus pyogenes* (Spy) Cas9 has been widely adopted as a platform for perturbing gene expression and protein levels in human cells (*1*). In this type II CRISPR system, the CRISPR-associated protein Cas9 performs targeted search and cleavage of double-stranded DNA guided by a CRISPR RNA (crRNA) that is complementary to the target sequence. The native CRISPR-Cas9 bacterial system has also been engineered to bind to DNA without inducing cleavage as a catalytically inactive Cas9 (dCas9). dCas9 has proven a powerful platform for modulating gene expression, particularly when fused to effector domains to permit perturbation of specific genomic loci (*2*).

Ideal gene editing or modulation tools require both high sensitivity (i.e., high probability of binding or cleavage at a targeted site) and superb specificity (i.e., low probability of binding or cleavage at nontargeted sites) (*3*, *4*). Because the biophysical processes involved in target search and binding necessarily underlie this sensitivity and specificity, they have been the subject of extensive investigation. Such work has revealed that the Cas9 ribonucleoprotein (RNP) first associates to an NGG protospacer adjacent motif (PAM) and then hybridizes to 8 to 12 target nucleotides abutting the PAM known as the “seed” region. Mismatches within this seed region inhibit stable RNP:target complex formation, whereas mismatches located distal to this region act to reduce the lifetime of RNP:target complexes (*5*). Building off of this work, and in combination with insights gleaned from characterizing Cas9 structures (*6*–*8*), others have characterized how DNA unwinding and subsequent conformational changes gate the activity of domains responsible for catalytic cleavage (HNH and RuvC) after binding (*9*–*11*). Lastly, recent work has suggested that Cas9 RNP:target interactions proceed along multiple paths, some of which may pass through or terminate in nonproductive states that limit Cas9 activity (*10*, *12*, *13*).

Thus, while the steps of canonical Cas9 binding are known, principles underlying sequence-dependent efficacy across guide sequences and sequence-dependent sensitivity to single-guide RNA (sgRNA):target mispairing given a guide sequence have been less comprehensively addressed. Most biophysical studies have measured relatively few RNP:target pairs, and while recent work has extended the number of off-target binding measurements per guide, the total number of sgRNAs profiled remains limited (*14*, *15*). Furthermore, even scalable technologies for measuring DNA-protein interactions, such as HiTS-FLIP (*16*), HT-SELEX (*17*), Bind-n-seq (*18*), BET-seq (*19*), and BunDLE-seq (*20*), often have limited kinetic resolution, and most of these methods are ill-suited to measuring either transient or low-affinity interactions, complicating comprehensive inference of off-target activity. The lack of diverse biophysical data across many guides and many off-target sites leaves few avenues for modeling Cas9 off-target activity (*21*, *22*).

To measure Cas9 binding in a quantitative and scalable manner, we developed a massively parallel nitrocellulose filter-binding assay by replacing autoradiography with a sequencing-based readout, enabling a label-free measurement of dCas9 RNP binding kinetics to thousands of off-targets in a single experiment (*15*). Here, we further optimize and parallelize this filter-binding technique and generate binding and cleavage data for more than 45,000 on- and off-target DNA sequences across 90 distinct sgRNAs. In so doing, we more than double the number of publicly available off-target binding measurements. Our data highlight the diversity of RNP biochemical behavior when loaded with different sgRNAs: Some sgRNAs are highly specific and exhibit large changes in binding when mismatches are present at the concentrations probed, while others are much less sensitive to mismatches. We demonstrate that context sequence outside the target and PAM can significantly modulate RNP association rates, which are correlated with Cas9 targeting efficacy in cells. Lastly, we develop a predictive biophysical model for Cas9 binding and cleavage of off-target sites.

## RESULTS

### Massively parallel filter binding enables scalable, quantitative measurement of Cas9 binding

**Fig. 1:**
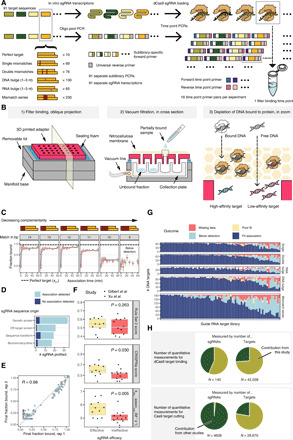
Kinetic profiling of dCas9 binding by massively parallel filter binding. (**A**) Experimental overview: Cas9 targets designed to 90 sgRNAs are synthesized on an oligonucleotide array. Ninety-one distinct sublibrary primers amplify each sgRNA’s targets separately. Each library is barcoded with forward and reverse barcodes in a second PCR. Filter binding is performed, and all time points and sublibraries are pooled for sequencing. (**B**) Three views: (1) A plate adaptor sits on a 96-well plate vacuum manifold; (2) sample is passed through the nitrocellulose membrane and collected in a deep 96-well plate; (3) sample passes through the nitrocellulose, retaining bound DNA in the membrane. (**C**) Example association curves for λ1 off-target sequences. Yellow bars signify 90% confidence intervals. Dashed line signifies perfect target final fraction bound. (**D**) Summary of sgRNAs included in study. (**E**) Reproducibility of λ1 targets’ final fraction bound across two sublibraries and sgRNA preparations. Bold gray lines (lower left) denote limits of detection. (**F**) Comparison of “Rule Set 2” scores, CRISPRia, and on-rates in discriminating effective versus ineffective sgRNAs, as determined by genetic screening. (**G**) Summary of association curves for all studied sgRNA sublibraries. (**H**) Pie chart demonstrating scale of presented data relative to other published studies of Cas9 off-target activity.

We first selected 90 gRNA sequences and designed a matched library of approximately 600 DNA targets for each of these guides (one “sublibrary”). gRNAs were curated from a variety of sources, including genetic screens, Cas9 off-target screens, and efforts to characterize Cas9 biochemistry. Sequence transformations of the curated sequences were also included. Sequence transformations consisted of taking the complement, reverse, or reverse complement of parts of gRNA sequences to allow direct comparison between nucleotide composition-matched gRNA sequences. Each sublibrary included DNA targets with all single mismatches; 66 contiguous double mismatches; 10 noncontiguous double mismatches; all single RNA:DNA bulges plus select double and triple bulges; 230 contiguous mismatch series consisting of rA:dA, rC:dC, rG:dG, and rU:dT mismatches from a start to end position; and 12 fixed sequences common to all sublibraries. In total, 54,349 targets for 91 sublibraries (including a duplicate sublibrary for the λ1 sgRNA) were designed (table S1). For each sublibrary, a corresponding sgRNA was prepared and loaded in dCas9, while the DNA was divided and barcoded with 16 distinct time point primers using polymerase chain reaction (PCR) (Fig. 1A) to allow quantification of a binding time course (see Materials and Methods).

We next designed a massively parallel filter-binding apparatus to permit processing binding time courses of sublibraries in 96-well plates (Fig. 1B). As part of this workflow, we use a nitrocellulose membrane to bind to protein-bound DNA targets and then collect and sequence the unbound DNA in the filtrate, thereby quantifying binding through measurement of depletion. The nitrocellulose membrane is placed over a three-dimensional (3D) printed 96-pronged adapter engineered to mate with a 96-deep well plate. When the specified association time for a sublibrary time point elapses, the sample is applied to the membrane and flow-through collected by vacuum filtration. At the end of the experiment, filtrate from every well is pooled and sequenced. Relative to our previous protocol (*15*), this 96-well design required 70% less hands-on time, 90% less reaction volume, and 85% less in cost.

Target counts from sequencing data were fit to a one-step association model (see Materials and Methods), resulting in two fit parameters: final fraction bound (*f*_final_) and an observed rate (*k*_obs_). Confidence intervals for each time point were constructed assuming Poisson noise. Targets for which the first time point neared the binding level of the last time points were fit to final fraction bound only. Furthermore, targets wherein time points’ confidence intervals for estimated fraction bound overlapped zero were separately flagged as binding below our detection limit (see Materials and Methods). Off-targets with an extreme fit rate or final fraction bound were flagged as poor fits. Some targets could not be fit, usually because of very low counts, and were labeled missing data.

We first conducted filter-binding association experiments on all 91 sublibraries with 5 nM dCas9 RNP and 100 pM total DNA. Overall, fit association curves for fraction bound generally fell within count-based confidence intervals across all time points. Inspecting the results showed patterns consistent with past work on dCas9 targeting rules, including 5 to 10 base pairs (bp) of sequence complementarity comprising a seed sequence that is sufficient for binding at 5 nM RNP (Fig. 1C) (*23*, *24*). Twelve of 91 (13%) perfect target sequences could not be fit, largely because binding levels were below the threshold of detection. Observation of detectable binding activity depended on the origin of the curated sequence (Fig. 1D). Half of the unfit sgRNAs contained 17 or more guanines/cytosine base pairs compared to 13% of fit sgRNAs (*P* = 2 × 10^−3^, binomial test). By screening sgRNA sequences in silico with RNAfold (*25*), another four exhibited extensive secondary structure (fig. S1A) that could interfere with the folding of sgRNA hairpins, a characteristic known to lead to poor sgRNA performance (*26*). Across the 79 sgRNAs with valid perfect target measurements, binding to 29,232 target sequences was quantified (Fig. 1G and table S2), substantially more off-target measurements than previous efforts (fig. S1B and Fig. 1H). An additional 5983 targets were classified as binding below our detection limit. Among these, the targets least likely to be quantified were designed to harbor RNA bulges or long series of mismatched bases. To quantify experimental variability, we prepared and assayed two separate sublibraries for the λ phage genome target known as λ1. We found that the fit final fraction bound was in good agreement across replicates (*R* = 0.98; Fig. 1E).

We next aimed to compare the parameters estimated from these experiments with in vivo activity scores. We first classified the subset of sgRNAs with published CRISPR interference (CRISPRi) activities as either effective or ineffective (*21*, *22*) and assessed whether the two classes exhibited differences in fit biophysical parameters. As a baseline, we applied two published predictive algorithms for CRISPRi guide activity: Rule Set 2 (*3*) and the CRISPRia model scoring (table S3) (*22*). Scores reported by both methods were higher for effective than ineffective sgRNAs (Fig. 1F). However, differences in Rule Set 2 scores were not significant, while CRISPRia scores met statistical significance (*P* = 0.030, Wilcoxon rank sum test). We then compared the discriminative power of our quantified *k*_obs_ and *f*_final_. Final fraction bound for on-target sequences mostly exceeded 50% and did not correlate with guide efficacy. In contrast, association rates for effective sgRNAs were significantly faster than those of ineffective sgRNAs (*P* = 0.005, Wilcoxon rank sum test). This observation is consistent with recent CRISPRi data demonstrating that apparent association rates govern CRISPRi activity in human cells (*27*).

### High-throughput kinetic measurements reveal diverse sequence landscape of dCas9 association

**Fig. 2:**
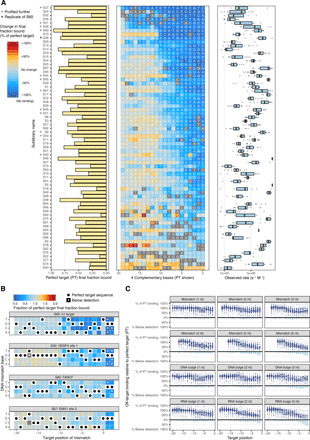
Diversity of dCas9 off-target association across sgRNA. (**A**) Association data for sgRNA from zero (PAM-proximal) to perfect (PAM-distal) complementarity. The perfect target final fraction bound is shown for each sgRNA on the left; off-target binding relative to perfect target is shown in the middle; the distribution of observed on-rates for the 20 targets in the center column is shown on the right. (**B**) Single mismatch data for four sgRNAs. PAM mutations are often near or below the limit of detection (asterisks), but many seed mismatches (positions −8 to −1) are within dynamic range. (**C**) Summary of association data collected across measured sgRNAs. Each panel visualizes mismatch or bulge series (of varying size) positioned alongside each base pair of the target sequence. Above the line, binding is reported as a percentage of perfect target binding level. Below the line, bars summarize the percent of sgRNA for which the off-target binding was below detection. For example, the majority of sgRNAs exhibit undetected binding for 3-bp RNA bulges in the seed (bottom right facet).

To assess variation in dCas9 association across diverse sequences, we first visualized the distribution of *f*_final_ and *k*_obs_ for off-target sequences with series of 0 to 20 complementary nucleotides at the PAM-proximal end of the target (Fig. 2A). Observed association rates spanned a 30-fold range across perfect targets, but for a given sgRNA, off-target association rates usually fell within a narrow range. Most sgRNAs showed little or no decrease in the final fraction bound (at 5 nM loaded Cas9) until complementarity dropped below 12 bp. However, some sgRNAs exhibited large decreases in final fraction bound when as few as one or two distal mismatches were introduced.

Although the λ1 sgRNA has been the sgRNA of choice for characterizing Spy Cas9 RNPs, the biophysical properties of the λ1 RNP appear atypical: *f*_final_ and *k*_obs_ for the λ1 perfect target are among the largest of the sgRNAs we profiled. In addition, *f*_final_ for λ1 RNP declines especially steeply for off-targets with fewer than 11 bp of complementarity (Fig. 2A, sublibrary S60). Most RNPs exhibit final fraction bound that is near perfect target levels until complementarity drops to 8 or 9 bp. Declines in final fraction bound for λ1 single mismatch targets are also more extreme than for most sgRNAs. Some, such as FANCF and EMX1 site 3, are minimally perturbed by single mismatches in their targets, unless the mismatches disrupt the canonical PAM (Fig. 2B).

Across all sgRNAs, most RNA:DNA mismatches or bulges had small effects on final fraction bound (Fig. 2C and table S4). Single RNA:DNA mismatches had particularly modest impact, generally only visible in first seven positions of the seed. Curiously, the presence of multiple distal mismatches slightly increased the final fraction bound for many sgRNAs (Fig. 2A). Recent single-molecule studies suggest that distal mismatches decrease the fraction of RNP:target complexes in an unwound state even while stably bound (*10*, *28*), which could correspond to differences in complex stability or adherence to nitrocellulose. We also, we observed that the sensitivity of a target to perturbation (as ordered in Fig. 2A) inversely correlated with the number of internal PAMs contained within the target sequence (Spearman *R* = −0.31, *P* = 0.01).

We noted that designing a DNA bulge in the gRNA:target DNA complex perturbed binding nearly as much as designing a mismatch. Further investigation showed that DNA bulges that matched the identity of the PAM-proximal nucleotide fared better than bulges of nucleotides that did not match but were located at the same position (*P* = 6 × 10^−5^, one-sided Wilcoxon rank sum test). This DNA insertion preference was most prominent at end positions (−1, −18, and −19) or center positions (−8 to −11) (fig. S2A and table S5). Positions outside of these regions did not exhibit such bias whether tested individually or in aggregate. As expected, design of a DNA bulge at the −1 position for targets with GGG PAMs, where a canonical PAM would be maintained, exhibited far greater binding than targets with HGG PAMs, where PAM usage would require a DNA bulge. Deletion of DNA target bases, which is expected to lead to the formation of an RNA bulge, typically led to a greater decrease in the final amount of target binding. For 3-bp RNA bulges in the 5 bp nearest the PAM, the majority of off-targets were at or below the limit of detection.

Having characterized off-target binding to naked DNA, we next asked whether the final fraction bound (*f*_final_) for a given sgRNA might be an accurate proxy for CRISPRi silencing capability. We first assessed predicted sgRNA CRISPRi activity for mismatched targets using a recently published model for CRISPRi efficacy at mismatched sites (*27*) by comparing *f*_final_ measurements to predicted activities for the λ1 sublibrary. We found that the vast majority of off-targets fell into one of two categories: low (<10%) predicted activity and low *f*_final_ (<30%), or moderate to high (>10%) predicted activity and high *f*_final_ (>60%) (fig. S2B), meaning that our biophysical measurements generally agreed with prior modeling efforts (Spearman *R* = 0.711, *P* = 4 × 10^−23^); furthermore, across all sublibraries with at least 50 *f*_final_ measurements, the correlations between these two metrics were overwhelmingly positive (65 positive of 69 tested, mean Spearman correlation of 0.453; fig. S2C and table S6).

### Cleavage assays highlight persistent subsaturating activity of Cas9 RNPs

**Fig. 3:**
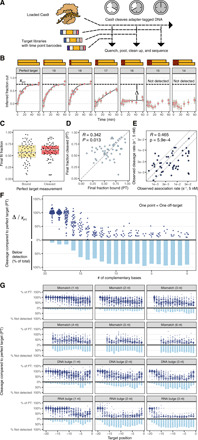
Matched cleavage data for Cas9 off-target libraries. (**A**) Cas9 cleavage experiments consist of time points read out in barcoded libraries in a PCR-free sequencing library. (**B**) Example cleavage data demonstrating cleavage as a function of base pairs of complementarity. (**C**) Final binding and cleavage levels for perfect targets are widely distributed. (**D**) Final binding level (*x* axis) is moderately correlated with final cleavage level (*y* axis) for perfect target sequences. (**E**) Joint distribution of 5 nM Cas9 association and cleavage rates. Solid lines show twofold changes (roughly the error of the assay). (**F**) Summary of extent of cleavage across 61 sgRNAs from 0 to 20 bp of complementarity, relative to perfect target cleavage level. Cleavage level drops steeply with fewer than 17 complementary bases. (**G**) Summary of extent of cleavage for other mismatch and bulge series across the length of the target.

To investigate sequence dependence of cleavage globally, we used the same barcoded 91 sublibraries to collect time point–resolved cleavage data at 5 nM active Spy Cas9 (Fig. 3A). Instead of passing samples through a nitrocellulose membrane, samples were quenched with EDTA and heat-inactivated. Cleaved products were left unamplified during sequencing. The prebarcoded libraries were directly sequenced without PCR amplification, and resulting counts were used to determine the observed cleavage rate and final fraction cleaved (Fig. 3B).

Notably, virtually all perfect targets fell short of 100% cleavage (Fig. 3C) even after incubating for an hour. While the dCas9 final fraction bound (*f*_final_) for perfect targets might be expected to fall significantly below 100%, the cleavage of template by active Cas9 over time would be expected to drive the reaction to completion in the limit of long incubations times. This subsaturating behavior (*29*, *30*) might be explained by Cas9 RNP binding to a target and, with some nonzero probability, entering a state where cleavage cannot occur and protecting the target. In general, the fraction of target bound exceeded the fraction cleaved (Fig. 3C), supporting such a hypothesis. We also observed that, among perfect targets, final cleavage levels weakly correlated with final binding levels (*R* = 0.342, *P* = 0.01; Fig. 3D), suggesting that some of the variation in cleavage fraction may stem from variation in final binding levels, but that the remainder is attributable to other sequence-dependent factors.

Other biochemical studies have concluded that cleavage is fast relative to rates of association (at 5 nM Cas9) for perfect targets (*12*, *13*). If cleavage were fast relative to association, then we would expect a high degree of concordance between the observed association and cleavage rates for perfect targets because Cas9 association ought to be the rate-limiting step in both cases. We compared observed cleavage and association rates for perfect targets and found that cleavage rates were only moderately correlated with association rates (*R* = 0.465; Fig. 3E). For many guides, we observe that perfect target cleavage is fast relative to association. However, a substantial fraction of guides induce cleavage more slowly than they associate, indicating that, for some guides, cleavage is slower than Cas9 association (at 5 nM).

Previous work has shown that cleavage is much more sensitive to imperfect matches than is binding (*23*) due to a conformational change required for target DNA cleavage (*7*, *31*, *32*). Our data are consistent with these findings. Across all sgRNAs, more than 85% of targets with 17 bp of complementarity exhibited detectable cleavage (Fig. 3F). Additional mismatches substantially decreased the fraction of targets cleaved: 38% of targets with 16 bp of complementarity exhibited cleavage below the threshold of detection, as did 62% of targets with 15 bp of complementarity (Fig. 3F). In contrast, for most sgRNAs, we observed only small changes in the final fraction bound for targets containing 15 bp of complementarity (Fig. 2A).

The off-target cleavage data revealed an important trend: Most targets with 15 or 16 bp of complementarity exhibited an intermediate level of final cleavage. In other words, off-target cleavage rates did not simply distribute near 0 (cleavage incompetent) and 1 (cleavage competent) but were instead broadly distributed (Fig. 3G). The existence of a single mismatch or DNA bulge in any position had a modest impact on final cleavage levels. In addition to targets with less than 17 bp of complementarity, targets with RNA bulges of 2 or 3 nucleotides (nt) at positions −1 to −17, as well as targets with contiguous mismatches of four or more base pairs at any position, exhibited little cleavage despite high levels of final fraction bound (table S7).

### Target context modifies rate of Cas9 association and cleavage

**Fig. 4:**
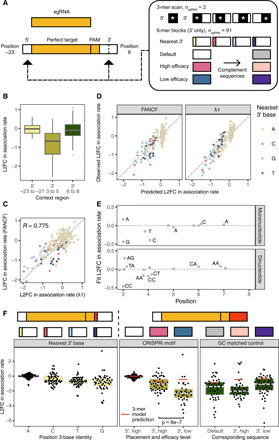
Impact of 5′ and 3′ sequence variation on perfect target binding and cleavage. (**A**) The 5′ and 3′ sequence variation was tested for all 3-mer blocks located beside two perfect targets as well as alternate base immediately downstream of the NGG PAM and specific 6-mer blocks 3′ for 91 perfect targets. (**B**) Impact of sequence variation 5′ of target sequence (positions −23 to −21) and 3′ of target sequence (positions 3 to 8), split by region. (**C**) Comparison of 5′ and 3′ sequence variation effects from 3-mer scan for FANCF and λ1 sgRNAs. (**D**) Results of learning a LASSO dinucleotide model for predicting the effect of 5′ and 3′ dinucleotides on association rate. (**E**) Visualization of the selected coefficients from the LASSO dinucleotide model, with high-weight features labeled. (**F**) Effect of 3′ sequence variation on association rates across 91 RNPs. In the left facet, the nucleotide flanking the NGG PAM downstream is shown to affect on-rate. In the center facet, extended motifs taken from a study of CRISPRi efficacy are shown. In the right facet, GC-matched 3′ sequence variants demonstrate that motifs are not driven solely by GC content.

In addition to assaying the 91 sublibraries described above, we also constructed two “3-mer scanning libraries” to test for the effects of flanking sequence on association and cleavage for λ1 and FANCF sgRNAs. These libraries were designed to harbor all possible trimers spanning the 5′ and 3′ flanks of the 23-bp target, extending 3 nt 5′ (to position −23) and 6 nt 3′ (to position 8) (Fig. 4A). Binding data from this library revealed that only sequence variation near the 3′ end of the target site reliably produced large (>2-fold) changes in the association rate of dCas9 (Fig. 4B) and that association rates for targets with variation either 5′ of the target site or more than 3 bp from the NGG PAM rarely differed from the rate for the default flanking sequence (Fig. 4B). While the typical association rate of Cas9 loaded with FANCF sgRNA was around a quarter that of a λ1 RNP, the effect of arbitrary 3′ nucleotides on the fold change of association rate relative to the default sequence context generally agreed, suggesting that these context-specific effects on association are guide independent (*R* = 0.775; Fig. 4C and table S8). The identity of the base nearest the PAM was the most important feature governing cleavage rates, consistent with a previously reported NGGH motif for Cas9 (*33*). Relative Cas9 observed cleavage rates correlated with relative association rates (fig. S3 and table S8), suggesting that, at 5 nM Cas9, cleavage of perfect targets is fast relative to association for all flanking sequences for both tested sgRNAs. Thus, while flanking sequences modify the rate of stable association, this assay lacks the time resolution necessary to assess effects downstream of association.

We next attempted to model the impact of 6 bp of 3′ sequence variation on relative association rates for both FANCF and λ1. We converted these measurements of context effects into a matrix of dinucleotide features (104 total features) and measured log_2_ fold changes relative to the default perfect target sequence for each guide. An additive model fit by LASSO regression captured most of the variance (cross-validated *R*^2^ = 0.731, *N* = 411; Fig. 4D). The fit parameters indicate that the presence of a G at the nearest 3′ position (NGGG-extended PAM) slows association, in this case by 27% (table S9). However, as suggested by an analysis of CRISPRi/a data (*22*), an extended PAM consisting of a 3′ CC (NGGCC) slowed the association rate even more. When combined with an additional 3′ C (NGGCCC), the model predicted over a twofold drop in association rate, more than double the reduction predicted for an NGGG-extended PAM.

Context variants were also included in the 91 sublibraries to assess the guide independence of effects of 3′ extended PAMs on association rate across a large number of guide sequences. To maintain library compactness, we tested the effects of five alternate 6-bp 3′ sequences and all three 1-bp substitutions downstream of the target on association to a perfect target sequence (Fig. 4A). We chose the 6-bp blocks that exhibited the most (NGGCGGGAG) and least (NGGGAATTT) CRISPRi activity in the study of Xu *et al*. (*21*) as well as complemented sequences to test whether association preferences were driven by the GC content of the sequence blocks (Fig. 4A).

Across context variants of all guides, association rates were typically the slowest to targets containing a G at the nearest 3′ base, consistent with an NGGH-extended PAM motif for achieving the most rapid association (Fig. 4F and table S10). While the median drop in association rate to an NGGG-extended PAM was 1.7-fold, inserting the CRISPRi-disfavored sequence produced an even larger (sixfold) reduction (*P* = 8 × 10^−7^ versus favorable, Wilcoxon rank sum test). This effect is not only due to GC content, as GC-matched controls showed smaller changes in their median relative association rates (two- to fourfold decreases). The unexpectedly large association rate decrease observed for this unfavorable 6-mer block was poorly predicted by the model trained on the scanning 3-mer data, suggesting that interactions beyond neighboring context nucleotides affect association rates. We also observed that association rates measured across perfect targets containing identical 6-bp blocks were more guide dependent and exhibited much larger variance than single base changes (1.8 versus 0.74 log_2_ fold units) (Fig. 4F). These observations suggest that aspects of extended PAM preferences are guide dependent and that while individual nucleotide changes have small effects, six or more nucleotide changes downstream of the PAM can lead to large differences in association rates for different sgRNAs.

### Cas9 concentration–independent mechanisms modify target binding and cleavage selectivity

Our initial survey of the binding of Cas9 loaded with 90 different gRNAs at 5 nM RNP affirmed two main points: The vast majority of library species exhibit intermediate levels of both binding (as measured by massively parallel filter binding) and cleavage. To determine whether these behaviors can be described by a simple two-state binding model and to quantify the presence of nonproductive bound states, we selected 12 of the 90 gRNAs for association profiling at 1.25 and 20 nM and cleavage profiling at 20 nM RNP.

Under a two-state binding model, the final fraction bound is a consequence of three independent parameters: protein concentration, *k*_on_, and *k*_off_. As protein concentration increases, the final fraction bound of a substrate also increases until it saturates at 100%. Yet, our extended dCas9 association data show that many Cas9 targets (e.g., a mismatch at position −4 for λ1 and a mismatch at position −4, −8, or −10 for ST3GAL5) do not saturate and instead plateau in their occupancy at levels far below 100%. Furthermore, for most sgRNA:target pairs, the observed final cleavage level was independent of Cas9 concentration (fig. S4A and table S11).

**Fig. 5:**
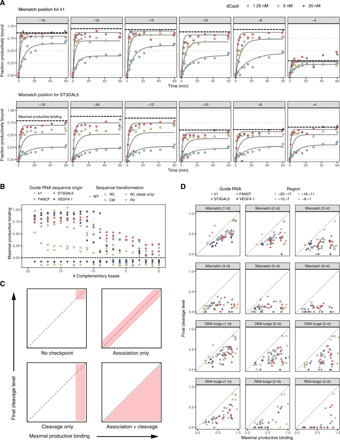
Joint fits for dCas9 association across three concentrations. (**A**) Joint fits for two sgRNAs and corresponding singly mismatched targets. The dashed line signifies the fit maximal productive binding. (**B**) Maximal productive binding is shown as a function of the number of complementary bases for all 12 jointly fit libraries. Most libraries show a transition around 10 bp of complementarity. Targets below the dashed line are below the limit of detection. WT, wild type; RC, reverse complement sequence; CM, complement sequence; RV, reverse sequence. (**C**) Expected joint distributions of maximal productive binding (*x* axis) and final cleavage level (*y* axis) under four different gating possibilities at Cas9 association and/or cleavage. (**D**) Observed joint distributions for maximal productive binding (*x* axis) and cleavage (*y* axis) for jointly fit libraries. With increasing perturbation of sgRNA:target matching, targets fall farther below the diagonal.

To address these discrepancies, we added an additional parameter to our fit capturing this “maximal productive binding” to allow saturation below 100% of the DNA targets present in solution. Fitting the data in this manner thus models two phenomena: concentration-dependent initial binding affinity and concentration-independent entry into a stable noncanonical bound state. Our data were generally well fit by jointly fitting the three concentrations (Fig. 5A and table S12), and these fits often returned maximal productive binding parameters well below 100%. We speculate that this subsaturating binding behavior may be due to a bound state not detectable by nitrocellulose-mediated filter binding, as documented previously for specific variants of LacR (*34*).

Among the 12 gRNAs we profiled, large differences in initial binding affinity (see Materials and Methods) were observed only for off-targets of λ1 Cas9 RNP (and RNPs derived from λ1 sequence transformations) (fig. S4B and table S13). Unlike the other tested sequences, λ1-derived sequences are devoid of internal, noncanonical PAMs. It is likely that RNP-PAM interactions can dominate the initial binding affinity observed for targets with multiple PAMs. In these circumstances, most target mismatches may not change the kinetics of binding because initial binding is mediated by PAM interactions rather than target complementarity. Our maximal productive binding measurement instead appears to align with the conventional understanding of Cas9 targets, which have an 8- to 10-bp seed region that is sensitive to disruption, an 8- to 11-bp PAM-distal region that is largely resilient, and an intermediate zone sensitive to large perturbations (Fig. 5B).

Because many targets appear incapable of attaining 100% productive binding, we hypothesize that there exist checkpoints in the binding process that can arrest Cas9 RNPs in nonproductive states. To understand the implications of our observations, we consider four possible models that either allow or disallow nonproductive states to trap target sequences prior to productive binding or cleavage. We explore the implications of these models under saturating protein concentrations ([Cas9] >> *K*_d_) (Fig. 5C). In the simplest model, with no gating, Cas9 associates to a target in a single step and executes cleavage to completion. Under this model, all target sequences would cluster around 100% productive binding and cleavage. Under the second model, the addition of a cleavage checkpoint irreversibly halts or prevents cleavage of some targets, preventing 100% cleavage even with increased protein concentration or time. When we instead model a nonproductive, nitrocellulose binding–incompetent interaction, we expect identical subsaturating behavior to arise in both association and cleavage data: RNP:target complexes forming nitrocellulose binding–incompetent interactions are prevented from progressing to cleavage, and all other targets are cleaved. Under our final model, gating occurs at both steps such that final cleavage levels are bounded by the maximal productive binding level, which may, in turn, range from 0 to 100% (area below the diagonal in Fig. 5C, bottom right). This final model can produce twofold subsaturating behavior, at both binding and cleavage stages.

We integrated the maximal productive binding estimates from our joint association fit data with the final cleavage level estimates from our 20 nM cleavage data to investigate the likelihood of each of the models described above. For a wide assortment of off-target sequences, the distribution of fit values strongly favors a model with subsaturating behavior of Cas9 for both productive association and cleavage (Fig. 5D). Targets with single RNA:DNA mismatches appear to exhibit extensive gating of productive binding, as measured by filter binding, but, of the fraction that appears bound, nearly all is able to cleave. Association and cleavage data for all other classes of off-target sequences are consistent with subsaturating binding and cleavage. For most Cas9 RNPs, the extent of association gating was bimodally distributed, which was not observed at the level of cleavage.

### Reversibility of Cas9 association declines over time

**Fig. 6:**
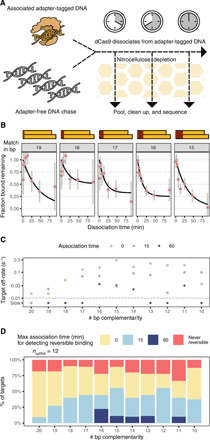
Quantification of dCas9 dissociation. (**A**) Dissociation experiments rely on addition of a DNA sink that blocks further binding to adapter-tagged libraries after initial variable-time incubation with 20 nM dCas9 RNP. (**B**) Fraction bound to λ1 targets as a function of time and the number of PAM-distal mismatches. Fraction bound is normalized to that of the first time point after addition of the DNA sink. (**C**) The observed λ1 sgRNA:dCas9 dissociation rate is shown as a function of the extent of complementarity (*x* axis) and the time elapsed before the addition of DNA sink (point color). Yellow points (0 association time) are taken from the joint association fits across three dCas9 concentrations. Points below the dashed line are below the limits of detection. (**D**) Summary of observable dissociation across 12 sgRNAs. With greater association time, fewer sequences exhibit dissociation. Fraction colored red has fit off-rates below 0.01 per second in joint association fits.

We previously noted that longer RNP incubation times ultimately led to reduced dissociation (*15*). To characterize this phenomenon across diverse guides, we collected dissociation data series after 15 and 60 min of association with 20 nM dCas9 in a manner analogous to the association experiments (Fig. 6A). We confirmed that chase DNA with 20 nt downstream of the PAM (no flow cell adaptors) was sufficient to quench dCas9 binding (fig. S5). Chase DNA was added to association reaction pools, which were then transferred to the nitrocellulose-covered vacuum manifold. λ1 targets with loss of PAM-distal complementarity demonstrated dissociation on the time scale of minutes (Fig. 6B). As complementarity declined from 20 to 16 bp, the average off-rate for λ1 targets increased monotonically (Fig. 6C and table S14). Our results were thus in line with our previous study of λ1 targets (*15*).

Overall, we tested 6865 off-target sequences across the same 12 sgRNAs measured across multiple dCas9 concentrations. Of these, 2300 did not have sufficient binding prior to dissociation, 618 were fit to a negligible off-rate in the joint association fit, and 2548 exhibited dissociation below the limit of detection given the time scale for the dissociation experiments we conducted. The remaining 1399 off-targets spread across the 12 gRNAs exhibited a similar pattern as seen for λ1: The loss of complementarity PAM-distally, from 20 to 16 bp, increased the observable dissociation, from 9 to 44% (for 15-min association experiments) and from 0 to 22% (for 60-min association experiments) (Fig. 6D). This increase in the fraction of RNP:target complexes capable of releasing targets with PAM-distal mismatches supports the hypothesis that full target:guide pairing substantially reduces the reversibility of Cas9 binding.

### Cas9 binding and scission exhibit distinct sensitivities to target perturbation

**Fig. 7:**
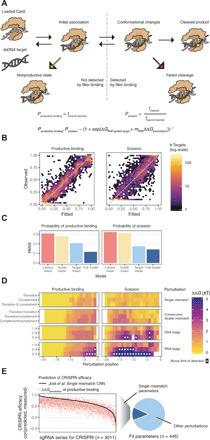
Model for Cas9 association and cleavage. (**A**) Illustration of checkpoints for productive association and successful cleavage. Reverse reactions from these nonproductive states are slow. The probability of productive binding (*P*_productive binding_) is equal to estimated maximal productive binding. The probability of scission (*P*_scission_) is equal to the final fraction cleaved divided by maximal productive binding. The full biophysical model for *P*_productive binding_ and *P*_scission_ as a function of ΔΔ*G*_perturbation_, *ΔG*_RNP:perfect target_, and *m*_RNP_ is shown. (**B**) 2D histogram of the fitted and observed probability of productive binding and scission estimates across all Cas9 RNPs. (**C**) The RMSE in predicting *P*_productive binding_ and *P*_scission_ is shown for our full model and alternative simpler estimates. (**D**) Fit ΔΔ*G*_perturbation_ values for select targets shown for productive binding and cleavage, plotted by perturbation position (*x* axis) and type (*y* axis). White circles represent measurements above the limit of detection. (**E**) Comparison of productive binding ΔΔ*G*_perturbation_ versus Jost *et al*. estimates for predicting relative CRISPRi activity within an sgRNA series (left). Breadth of target types scoreable with ΔΔ*G*_perturbation_ values relative to those scoreable by the Jost CNN (right).

Our results suggest a model wherein Cas9 traps off-target sequences in slowly acting or nonproductive states that both are not bound by nitrocellulose and block progression to cleavage (Fig. 7A). Under this model, two concentration-independent parameters determine whether cleavage will occur at a target site when saturated with protein: the probability of productive binding and the probability of scission (conditioned on productive binding). The probability that a Cas9 RNP:target interaction cleaves an accessible target is the product of the two.

To learn about the sequence determinants of productive binding and scission, we designed a biophysical framework adaptable to both parameters. We first assigned each of the 12 Cas9 RNP:perfect target pairs a baseline energy value to capture the partial productive binding and scission observed for perfect targets (Δ*G*_RNP:perfect target_). To group targets across Cas9 RNPs, we annotated mismatches (transition, complement, or both) and RNA and DNA bulges (from 1 to 3 nt) at each position for each RNP:target pair and defined targets with identical annotations as sharing the same “target perturbation” (tables S15 and S16). We then fit an energy penalty that decreases the likelihood of productive binding or scission (ΔΔ*G*_perturbation_) to every target perturbation. Initial attempts at modeling suggested that different Cas9 RNPs exhibited differential sensitivity to sequence perturbations; for this reason, we also included an energy scaling parameter (*m*_RNP_) that allowed the overall magnitude of these energy perturbations to vary by guide.

Leave-one-out cross validation of RNP datasets suggested that perturbation penalties were stable and well correlated with maximal productive binding estimates on held-out data (mean Spearman correlation of 0.81; fig. S6A). Using this framework, we fit productive binding energy penalties for 446 distinct target perturbations and both RNP-specific energy parameters for 11 dCas9 RNPs using 4871 binding measurements, and, separately, scission energy penalties for 439 perturbations and RNP-specific energy parameters for 10 Cas9 RNPs using 3603 binding and cleavage measurements (see Materials and Methods; Fig. 7B and table S17). The logarithm of the productive binding energy scaling parameter was highly correlated with RNP:perfect target binding baseline energy (*R* = −0.78, *P* = 7 × 10^−3^; table S18), as was the logarithm of the scission energy scaling parameter (*R* = −0.78, *P* = 8 × 10^−3^; fig. S6B). From this, we infer that more energetically favorable Cas9 RNP:perfect target pairs suffer commensurately larger penalties when mismatches disrupt their pairing, unexpectedly linking binding sensitivity to specificity.

Biophysical modeling demonstrated improved performance over taking the mean value per perturbation as measured by root mean square error (RMSE), especially for productive binding (productive binding RMSE of 0.12 versus 0.21; scission RMSE of 0.14 versus 0.17; Fig. 7C), and produced clear insight into how mispairing between sgRNAs and DNA targets influences productive binding and scission probability. Sequence perturbations at PAM-distal positions (−20 to −13) were universally assigned no energy penalty, and a 7-bp seed match was sufficient to discern some level of productive binding. Furthermore, productive binding loss due to PAM-proximal seed mismatches could be partially rescued by increasing complementarity PAM-distally, requiring approximately two additional distal matches to compensate for each seed mismatch (Fig. 7C and fig. S6C). In contrast, scission is most perturbed by mismatches spanning positions −16 to −11 (Fig. 7D), and a series of six or more mismatches anywhere between sgRNA and target usually abrogate scission activity (fig. S6C). These findings are consistent with studies of truncated gRNAs that suggest that Cas9 RNP binding is stable with approximately 14 nt of gRNA complementarity (*32*, *35*).

We also explored whether maximal productive binding energy penalties for doubly mismatched targets were additive with respect to their constitutive single mismatches. Consecutive double mismatches clearly diverged from additivity. Most notable were PAM-distal positions where single mismatch energy penalties were set to 0, but double mismatch energy penalties exceeded 1 *k*T (fig. S6D and table S19). In contrast, nonconsecutive double mismatches that were at least 4 nt apart appeared additive, suggesting that sufficiently distant mismatches may have independent effects on productive binding.

Lastly, we evaluated whether productive binding ΔΔ*G*_perturbation_ values could predict relative CRISPRi knockdown in human cells. Across 3011 promoters with singly mismatched sgRNA series, we compared measured CRISPRi phenotypes both to our estimated ΔΔ*G*_perturbation_ of productive binding and to activity predicted by a convolutional neural network (CNN) that incorporated additional features beyond the identity of the RNA-DNA mismatches (such as GC content and position relative to transcription start site) and was trained on this dataset (Fig. 7E) (*27*). The mean Spearman correlation with measured CRISPRi activity was 0.508 for ΔΔ*G*_perturbation_ versus 0.667 for the CNN. Thus, while a CNN specifically trained on these data outperformed our mismatch-only model, overall scores were remarkably similar (mean correlation between the models was 0.74), suggesting that biochemical parameters governing Cas9 binding and cleavage are the dominant features influencing in vivo efficacy. However, because the CNN model was trained only on single mismatch data, it is unable to predict more complex perturbations, whereas our ΔΔ*G*_perturbation_ predictions span a broad variety of off-targets including 1 to 3 nt bulges and mismatch series of arbitrary size that greatly expand the scope of off-target assessment. Unexpectedly, 204 of the 386 more complex perturbations we estimate (53%) have predicted off-target activity at least as great as that predicted for a singly mismatched target, highlighting the vital importance of considering off-targets with these and similar perturbations in in vivo off-target assessments.

## DISCUSSION

Here, we present a large corpus of Spy Cas9 binding and cleavage across diverse sgRNA sequences and corresponding DNA off-targets enabled by further parallelizing our pooled, sequencing-based filter-binding assay. We now report an amortized cost of 8 cents per off-target measurement. In contrast to imaging-based methods that require maintaining fluidics and microscopes, our new design requires minimal equipment: principally, a single 96-well vacuum manifold. We profiled ~10^3^ off-targets per RNP per experiment and speculate that future applications of this technology to Cas9 or other DNA- or RNA-binding proteins of interest could assess more than 250,000 targets with straightforward protocol modifications. Thus, we believe that massively parallel filter binding represents cost-effective and operationally straightforward tool for profiling protein–nucleic acid binding kinetics.

In this study, we show that differences in perfect target association kinetics appear to explain some of the differences in screening efficacy across sgRNAs. Two underappreciated phenomena—sgRNA folding and disadvantageous extended PAM sequences—appear to modify efficacy at the level of binding, with implications for both CRISPRi/a and CRISPR KO (knockout) screens (*26*). We then compared perfect target biophysical measurements to CRISPRia scores for sgRNAs with CRISPRi measurements (*22*) and found that empirical measurements of RNP association exhibited greater predictive power for sgRNA efficacy. As a filter-binding experiment is generally simpler and faster than a CRISPRi-based screen in cells, we believe that measurement of association rates in vitro may be a useful alternative to computational and cell-based methods for evaluating guide efficacy. These findings also reiterate the importance of active-site titration to disentangle the numerous factors that can interfere with CRISPR enzyme activity in vitro (*12*).

Yet, altered Cas9 RNP association to off-targets does not appear to explain reduced activity at off-target sites. We observed that association kinetics of off-target sequences usually cluster tightly around that of their respective perfect targets. Thus, changes in off-target activity are unlikely to be governed by differences in cleavage or association rate, consistent with proposed mechanisms of high-affinity binding for other nucleic acid–guided proteins (*36*). This contrasts with the role of increased Cas9 off-rate in explaining lower off-target activity, as in the case of PAM-distal mismatches (*15*). Furthermore, initial binding affinity suggested that little more than a few PAMs might be adequate for appreciable dCas9 occupancy. Both observations appear in conflict with the high reported specificity of sgRNAs in CRISPRi screens.

We also observe that scission of many bound DNA targets is incomplete, supporting a branching rather than linear (*32*) binding and cleavage process involving intermediate states. Other investigators have attributed incomplete cleavage to the existence of a nonproductive state comprising 15% of the RNP:target complex and slow biphasic reaction steps in Cas9 catalysis (*10*, *12*, *13*). The expanded scope of our off-target dataset strongly suggests that the probability of cleavage varies by both the sgRNA sequence and the extent of complementarity to the corresponding DNA target. The most likely explanation is that the probabilities of both stable binding and scission of stably bound targets are strongly dependent on the sequence identity of the RNP:target pair in a concentration-independent manner, ranging both above and below the 15% seen for commonly studied RNP:target pairs. This behavior suggests that multiple checkpoints have evolved to mitigate Spy Cas9 off-target activity independent of Cas9 RNP:target interaction affinity.

Unexpectedly, we show that Cas9 filter-binding experiments appear to reflect additional state information beyond the binary notion of bound or unbound. Specifically, off-targets with numerous sgRNA:target mismatches are rarely fully depleted when passed through nitrocellulose, and increasing RNP concentration does not enhance depletion. The inferred maximal depletion for an RNP:target pair consistently serves as an upper bound for the fraction of target that can be cleaved, suggesting that this state is not competent for cleavage. We speculate that off-target sites trap Cas9 in slowly acting or nonproductive states that disassemble when passed through nitrocellulose (Fig. 7A). The molecular basis for such nonproductive states is left unaddressed by our filter-binding experiments. Discriminating between explanations such as partial R-loop formation and novel Cas9 conformations will require alternative methodologies that report on the spatial proximity of Cas9 RNP complex components at angstrom-scale resolution.

The manner by which Cas9 engages with on- and off-target sites has clear practical relevance for application of Cas9 technologies. A model of dCas9 binding similar to ours has been proposed to explain how mismatched sgRNAs can permit concentration-independent, “noiseless” CRISPRi-mediated gene silencing in bacteria (*37*). The authors observe that dCas9 resists eviction by RNA polymerase extension and blocks gene expression with a fixed probability, *P*(stop), that positively correlates with target complementarity. We speculate that *P*(stop) may be functionally equivalent to what we report as the probability of productive binding and that nonproductive Cas9 RNP:target interactions are easily dismantled by either colliding with RNA polymerase or passing through nitrocellulose. Thus, our work adds to the growing biochemical evidence of nonproductive bound states (*12*, *13*, *38*).

Understanding off-target association and cleavage may prove key to engineering workhorse variants of CRISPR enzymes. Most studies have focused on optimizing Spy Cas9 cleavage (*9*, *39*, *40*), and a recent article confirmed that the association kinetics for the most widely used engineered Cas9s do not differ from their wild-type counterparts (*41*). Yet, engineering efforts designed around Spy Cas9 binding have achieved greater on-target efficacy and specificity (*39*). More broadly, off-target detection methods have demonstrated substantial time- and concentration-dependent off-target activity (*42*, *43*), and kinetic partitioning has been found to underpin enhanced specificity of engineered Cas9 derivatives (*44*). For this reason, protein engineering efforts are unlikely to offer a single solution for experiments that operate over different time scales with different tolerance for off-target effects, and more advanced biophysical models for Cas9 activity remain a top priority.

Despite the efforts of several groups, predicting the kinetics and thermodynamics of binding for an arbitrary RNP complex to target sequences remains an outstanding challenge. Previously uncharacterized 3′ sequence requirements for Cas9 binding 14 nt downstream of the PAM were only recently uncovered (*45*, *46*), which we confirm (fig. S5). While initial RNA sequencing data showed little to no off-target activity of CRISPRi (*47*), new results from screens of noncoding elements in human cell lines (*48*) and screens of essential genes in bacteria (*49*, *50*) suggest that a variety of sequences remain difficult to target without the possibility of substantial off-target effects. Our observation that guide sequences exhibiting strong on-target binding typically have more selective binding behavior may also have implications for rectifying poor gRNA performance.

We expect future Cas9 binding and cleavage models to address the multiple stages of Cas9 target engagement in a manner robust to guide and target sequence. This includes inactive protein fractions attributable to nonfunctional protein, guide misfolding, and nonproductive states. Models of Cas9 binding and cleavage that parameterize the molecular progression from PAM association to R-loop formation and target cleavage continue to mature (*51*, *52*) and may illuminate how multiple mismatches impede Cas9 binding and cleavage as well as how Cas9 RNP complexes enter nonproductive states. We anticipate that the generation of large-scale data on off-target binding, as well as detailed thermodynamic modeling of potential binding and cleavage events, will become only more important as an increasing number of guide sequences are deployed for therapeutic applications.

## MATERIALS AND METHODS

### (d)Cas9 RNP preparation

sgRNAs were in vitro transcribed using the NEB EnGen sgRNA Synthesis Kit (catalog no. E3322S) according to the manufacturer’s instructions, starting with 0.15 reaction units per sgRNA and scaled up to 0.5 units as needed to generate sufficient material for each sgRNA. sgRNAs were purified using Agencourt RNAClean XP beads for the all-sgRNA round (part no. A63987) and Zymo RNA Clean & Concentrator-5 (catalog no. R1013) for additional syntheses. Cas9 and dCas9 were provided by the Doudna laboratory.

For loading, each sgRNA was incubated at 98°C for 1 min and slowly cooled to room temperature. dCas9 was diluted to 100 nM and incubated with an equal volume of sgRNA at 20% excess in 1× binding buffer [20 mM tris-HCl (pH 7.5), 100 mM KCl, 5 mM MgCl_2_, 5% glycerol, heparin (0.05 mg/ml), 1 mM dithiothreitol, and 0.005% Tween 20], a final working concentration of 50 nM. Loaded dCas9 was further diluted to attain the desired concentration (1.25, 5, and 20 nM) for association experiments and 20 nM for dissociation experiments in 1× binding buffer.

### Library design and preparation

Single mismatches, contiguous double mismatches, noncontiguous double mismatches, nucleic acid bulges, contiguous mismatch series, and common fixed sequences were designed by varying sgRNA target sequence programmatically by custom script. The 54,349 designed off-targets were deduplicated into 46,393 unique sequences that were each assigned an element id. For example, deletion of adjacent positions in a homopolymer yielded multiple annotations but only one element id.

In addition to the 23-bp target and 6-bp 5′ and 3′ flanking sequence contexts, each sublibrary of target elements was assigned a 13-bp primer binding site to be placed upstream for amplification (*45*). Primer binding sites with GG or CC dinucleotides were removed to prevent PAM-only binding. Universal adapters (17 and 18 bp) were added to each end of the construct to permit amplification of all oligos at once. Oligonucleotides were synthesized in a single pool by CustomArray on a 92,918 array (each sequence in duplicate) and PCR-amplified using NEBNext 2X master mix (catalog no. M0541L).

Following the initial amplification, each sublibrary was amplified with 16 distinct pairs of barcoded forward and reverse primers in separate reactions (98°C denaturation, 68°C annealing, and 72°C extension). PCR products were purified using AMPure beads and quantified with a Qubit dsDNA HS kit (catalog no. Q32854) before dilution to 1 nM total oligo working concentration.

### Massively parallel filter-binding experiments

Custom-designed adaptors for loading samples into a vacuum manifold were ordered online via 3D printing from 3D Hubs (ABS FDM, 40% infill, 200-μm resolution). Adaptor surfaces were sanded down with 300-grit sandpaper to remove striations left by 3D printing. Before use, surfaces were coated with a superhydrophobic residue to prevent sample loss to wetting of the surface. Rust-Oleum NeverWet (Amazon) step 1 was first applied in two to four short bursts of spraying and dried in a fume hood for 2 hours. One coat of step 2 was applied and then left to dry overnight. A second coating was applied the next day and fully dried before use. Hydrophobic residues remained intact for a week but deteriorated and required new coatings for peak performance if left for longer periods.

The filter-binding vacuum manifold system was assembled by inserting a sterile 1-ml deep 96-well plate into the bottom of a 96-well vacuum manifold, placing the upper half over the plate, layering a cut section of Fibre Craft foam (Amazon) over the surface of the plate, and adding the custom adaptor to reach into the wells. To prepare nitrocellulose, a precut membrane was soaked in binding buffer before transferring to the surface of the adaptor to create a vacuum-tight seal.

For association experiments, 1.25, 5, and 20 nM dCas9 (10 nM dCas9 for context experiments) was incubated with 16 barcoded libraries individually (final library concentration, 100 pM) in 40 μl of 1× binding buffer at room temperature (between 22° and 24°C), timed to yield measurements at 1, 2, 3, 4, 5.5, 8, 11, 15.5, 21.5, 30, 42, 59, and 60 min of association plus three zero time points. For dissociation experiments, 20 nM dCas9 was incubated with 14 barcoded libraries at room temperature for each of the above association times followed by addition of a final concentration of 40 nM competitor on-target DNA to yield measurements at 1, 2, 3.5, 7, 13, 25, 47, and 90 min of dissociation plus two predissociation samples and four zero time points.

Each association and dissociation time point reaction was passed through the nitrocellulose filter and flow-through collected from the corresponding wells. Samples for six sublibraries were pooled and purified using Qiagen MinElute columns. Libraries were quantified by Qubit dsDNA HS assay in the case of association experiments and by qPCR with a standard curve derived from a Qubit-quantified dsDNA library in the case of dissociation and cleavage experiments. All libraries were sequenced PCR free using Illumina NextSeq v3 chemistry with 2 × 75 reads.

### Cleavage experiments

Loaded active Cas9 was added to barcoded target libraries in binding buffer followed by quenching with 16 mM EDTA and placing on ice, timed as in the association experiments. Following EDTA quench, reactions were immediately incubated at 65°C for 10 min to deactivate Cas9. Reactions were pooled and cleaned up using Qiagen MinElute columns as above.

### Electromobility Shift Assays

DNA oligos were ordered from IDT such that a forward oligo with 6 bp of sequence upstream of the Cas9 target sequence partially overlapped reverse oligos with variable numbers of bases downstream of the target (7, 16, or 20 bp). A reverse, Atto532-labeled oligo that extended 20 bases downstream was ordered in parallel to permit visualization of results on a Typhoon imager. All reverse oligos were annealed and extended with the forward oligo using NEBNext 2X master mix. Labeled DNA was added to dCas9 RNP with or without unlabeled competitor DNA of uniform length present (again either 7, 16, or 20 bp downstream of the target), for final concentrations of 200 pM labeled DNA, 5 nM dCas9 RNP, and 20 nM competitor. Bound and unbound labeled DNA was separated by electrophoresis using Novex 10% TBE precast gels (catalog no. EC6275BOX).

### Sequence read data analysis

Fastqs were first trimmed of adapters using SeqPurge (*53*). Trimmed forward and reverse reads were merged using FLASH (*54*) with the max-mismatch-density parameter set to 0.01 and the min-overlap parameter set to 10. Merged fastq reads were assigned to target library sequences, permitting one single-nucleotide mismatch in the sublibrary primer sequence and an exact match throughout the rest of the target. Reads were aggregated by target to produce a count table of counts per target and time point.

### Estimating final fraction bound, final fraction cut, initial binding affinity, and maximal productive binding

For single concentration associations, count data for each target were fit to the following equation in R using the nls function

$$c(t)\sim c_{\text{control}}(t)\times \frac{c(0)}{c_{\text{control}}(0)}\times (1-f_{\text{final}}\times (1-e^{-k_{\text{obs}}t}))$$

*c*(*t*) is the target sequence count at time point *t*. *c*_control_(*t*) is the control sequence count at time point *t*. For all experiments, control sequence counts consisted of summing counts for fully complemented target sequences (“CM”) and targets with PAM GG dinucleotides replaced with TT dinucleotides (“KO”). *f*_final_ is the final fraction bound, and *k*_obs_ is the observed rate constant. *f*_final_ was initialized to 0.9 and *k*_obs_ to 0.024 per nM per minute times the Cas9 concentration. The control parameter was set to nls.control(maxiter = 300,warnOnly = TRUE).

For data visualization, fraction bound [*f*_bound_(*t*)] was inferred as follows

$$c_{\text{expected}}(t)=\frac{c(0)\times c_{\text{control}}(t)}{c_{\text{control}}(0)}$$

$$f_{\text{bound}}(t)=1-\frac{c_{\text{observed}}(t)}{c_{\text{expected}}(t)+0.1}$$

0.1 was added to the denominator to prevent divide-by-zero errors in the rare case of zero reads at time point 0. Confidence intervals (90%) for inferred fraction bound were calculated by adding and subtracting 1.64 times the square root of *c*(*t*) for each time point and calculating final fraction bound as before.

Some targets were not fit due to the following criteria:

1) Binding was not dynamic over the course of the experiment. The average final fraction bound at the two latest time point did not exceed the upper limit of the 90% confidence interval for each of the first two time points.

2) Counts for the target sequence were too small to be reliable. There were fewer than five time points in the first half of the experiment that exceeded 30 reads.

Targets that did not meet both of the above requirements were further stratified. Of the targets that were not dynamic, those that had at least five time points in the second half of the experiment where the entire 90% confidence interval exceeded 0 were fit to a horizontal line based on the time points in the second half of the experiment (no rate parameter). Of the remaining targets, those that averaged below 15% final fraction bound in the second half of the experiment were flagged as low affinity. The remainder (i.e., targets with large confidence intervals and small change in final fraction bound from the beginning to the end of the experiment) were annotated as noisy.

We observed that after performing initial fits, some time points were consistent outliers across DNA targets in the same experiment. This could be explained by a biased control target count for such outlier points, which would affect the inference of fraction bound for all other targets. To address this, time points for which the magnitude of the averaged residuals exceeded 2.5 times the median magnitude of the averaged residuals were excluded, and count data were refit using the remaining time points. An average of 1.9 time points out of 16 and a median of 1 out of 16 were excluded per experiment across all association experiments. The association rates we report refer to *f*_final_ times *k*_obs_. Cleavage data were fit in the same manner as binding data.

For joint association analysis across three dCas9 concentrations, measurements outside an inferred fraction bound from 0 to 150% were excluded as outliers. After filtering, the following equation was used

$$c(t,M_{\text{dCas}9})\sim c_{\text{control}}(t,M_{\text{dCas}9})\times \frac{c(0,M_{\text{dCas}9})}{c_{\text{control}}(0,M_{\text{dCas}9})}\times (1-J(t,M_{\text{dCas}9}))$$

$$J(t,M_{\text{dCas}9})=\frac{k_{\text{on}}\times M_{\text{dCas}9}}{k_{\text{on}}\times M_{\text{dCas}9}+k_{\text{off}}}\times (1-e^{-(k_{\text{on}}M_{\text{dCas}9}+k_{\text{off}})t})\times f_{\text{bound},\text{maximal}}$$

*M*_dCas9_ is the concentration (M) of dCas9. *f*_bound,maximal_ is the maximal productive binding.

For joint fits, *k*_on_ was initialized to 2 × 10^7^ per M per minute, *k*_off_ to 0.02 per minute, and *f*_bound,maximal_ to 0.85.

Initial binding affinity was calculated from *k*_on_ and *k*_off_ in the manner of a *K*_d_, reported in units of *k*T

$$\Delta G_{\text{initial binding}}=-\text{log}(\frac{k_{\text{off}}}{k_{\text{on}}})$$

As in the association experiments, targets in the dissociation experiments were required to exhibit a 15% drop in fraction bound over the course of the experiment to qualify (to be sufficiently dynamic). Sequences that did not start above 15% bound before dissociation were deemed low affinity and removed from consideration. Only time points following quench (*t* > 0) were included in the fit.

Dissociation experiments were fit to a distinct equation

$$c(t)\sim c_{\text{control}}(t)\times \frac{c(0)}{c_{\text{control}}(0)}\times (1-f_{\text{bound},\text{minimum}}-D(t))$$

$$D(t)=(f_{\text{bound},\text{initial}}-f_{\text{bound},\text{minimum}})\times e^{-k_{\text{off}}t}$$

*f*_bound,minimum_ is the fit fraction of dCas9 that did not reverse on the time scale of the experiment. *f*_bound,initial_ is the fit fraction of dCas9 bound at *t* = 0.

After fitting curves, fits with an *k*_obs_ or *k*_off_ below 0.02 per minute, an observed rate above 2 per minute, an *f*_final_ above 1.2, or an *f*_final_ below −0.2 were excluded as poor fits.

The LASSO model for 3′ context effects was fit using the R package glmnet. Coefficients for dimer identities by position were retrieved by running the coef command with parameter s = “lambda.1se”.

### Defining biophysical model parameters for productive binding and scission probabilities

We assume that the choice for a Cas9 RNP:perfect target pair between entering a productively bound state or a nonproductively bound state can be modeled with a simple energy gap: Δ*G*_RNP:perfect target_, whereby more negative energies favor productive binding. We anticipate that most Cas9 RNPs should have a value near or below zero such that the probability of productive binding is near or above 50%. In addition, we assign each sequence perturbation a fixed adjustment to the energy gap that applies independent of Cas9 RNP: ΔΔ*G*_perturbation_. However, it stands to reason that different Cas9 RNP:target pairings will be differentially affected by mismatches and bulges. Specifically, it is expected that RNP:target pairs that are more energetically favorable should conversely suffer larger energy penalties when disrupted. We first attempted a parameter-free correction by using DNA:DNA and RNA:DNA duplex hybridization energies estimated by MELTING5, but performance was poor. Instead, we introduced another parameter (*m*_RNP_) to scale ΔΔ*G*_perturbation_ per RNP.

From these parameters, we derived the probability of productive binding

$$P_{\text{productive binding}}={\left(1+\text{exp}(\Delta G_{\text{RNP}:\text{perfect target}}+m_{\text{RNP}}\Delta \Delta G_{\text{perturbation}})\right)}^{-1}$$

The same equation was used for probability of scission.

### Fitting biophysical model parameters for productive binding and scission

Maximal productive binding data were organized into a matrix of sequence perturbations by dCas9 RNP guide sequences. Maximal productive binding levels were subject to a series of quality control steps:

1) Overly large estimates of maximal productive binding (>150%) were replaced with missing values (NAs).

2) Alternate perfect target contexts were removed to ensure one value for Δ*G*_RNP:perfect target_.

3) Targets with slow initial *k*_on_ (<2,000,000 M^−1^ min^−1^) or fast initial *k*_off_ (>1 min^−1^) estimates were replaced with 2% maximal productive binding.

4) Targets with high estimates of maximal productive binding (>98%) were replaced with 98%.

5) Targets with low estimates of maximal productive binding (<2%) were replaced with 2%.

6) Sequence perturbations with fewer than four valid maximal productive binding estimates (from different RNPs) were removed.

After filtering, 465 perturbations remained for fitting, with 19 redundantly encoded perturbations. One RNP (the reverse complement of VEGFA site 1) was excluded entirely because of low levels of productive binding and few valid fitted values, leaving 11 columns for a 465 by 11 data matrix *d*.

All productive binding parameters were fit jointly using the nls.lm function in R. The geometric mean of the *m*_RNP_ parameters was constrained to 1 by fitting only 10 free parameters and inferring the 11th. *m*_RNP_ values were initialized to 1, and Δ*G*_RNP:perfect target_ values were initialized to 0. ΔΔ*G*_perturbation_ values were initialized by converting the difference in probability of productive binding from the perfect target to the perturbed target to a Δ*G* and taking the average across all dCas9 RNPs. If the average for a perturbation was below −0.1 *k*T, it was set to −0.1 *k*T.

*m*_RNP_ values were bounded between 0.2 and 5. Δ*G*_RNP:perfect target_ values were bounded between −6 and 3 *k*T. ΔΔ*G*_perturbation_ values were bounded between −0.1 and 6 kT.

The data matrix m was predicted as follows

$$\begin{array}{c} \hat{d}=1/(1+\text{exp}(t(\text{diag}(m_{\text{RNP}})\%*\%\text{matrix}(1,11,465)\%*\%\\ \text{diag}(\Delta \Delta G_{\text{perturbation}})+\text{diag}(\Delta G_{\text{RNP}:\text{perfect target}})\%*\%\text{matrix}(1,11,465)))) \end{array}$$

Residuals were reported to nls.lm by taking the difference between $\hat{d}$ and *d*, removing NA values, and converting the matrix to a vector. After fitting, ΔΔ*G*_perturbation_ values below 0 were set to 0.

After an initial fit, the top 20 perturbations ranked by mean absolute deviation were manually examined for potential outliers. Out of the 220 measurements examined, 9 dCas9 RNP:off-target pairs appeared to have extreme values and were designated as outliers. In a majority of cases, refitting the data had a marginal impact on the fitted values, suggesting that, overall, fits were robust to random error.

Leave-one-out cross validation was performed by removing one column from *d* and taking the Spearman correlation between learned ΔΔ*G*_perturbation_ values and estimated probabilities of productive binding (which would be unaffected by Δ*G*_RNP:perfect target_ or *m*_RNP_).

Probability of scission data was fit in much the same way, with some added steps and modifications. Measurements where the probability of productive binding was below 2% were replaced with NAs. Final cleavage levels above 99% were replaced with 99%. The probability of scission was calculated as maximal productive binding divided by final cleavage level. Probabilities of scission above 99% were replaced with 99%. Probabilities of scission below 10% were replaced with 10% because low levels of scission were difficult to resolve, especially when the probability of productive binding was low.

In total, 458 perturbations were able to be fitted, although one additional Cas9 RNP (reverse complement of the distal sequence of λ1) was removed because of low levels of cleavage across all targets. ΔΔ*G*_perturbation_ values were bounded between −0.1 and 7 kT because probability of scission for perfect targets generally exceeded the probability of productive binding for perfect targets, which increased the range of detection from Δ*G*_RNP:perfect target_ values. Lastly, out of the 200 measurements for the 20 perturbations with greatest error, only 4 measurements were deemed outliers.

## SUPPLEMENTARY MATERIALS

Supplementary material for this article is available at http://advances.sciencemag.org/cgi/content/full/7/8/eabe5496/DC1

## Appendix

View/request a protocol for this paper from *Bio-protocol*.
